# Predicting the Potentiometric Sensitivity of Membrane Sensors Based on Modified Diphenylphosphoryl Acetamide Ionophores with QSPR Modeling

**DOI:** 10.3390/membranes12100953

**Published:** 2022-09-29

**Authors:** Nadezhda Vladimirova, Elena Puchkova, Dmitry Dar’in, Alexander Turanov, Vasily Babain, Dmitry Kirsanov

**Affiliations:** 1 Institute of Chemistry, Saint-Petersburg State University, Peterhof, Universitetsky Prospect, 26, 198504 Saint-Petersburg, Russia; 2 Yu. A. Ossipyan Institute of Solid-State Physics, Russian Academy of Sciences, Chernogolovka, Moscow Oblast, Academician Osipyan Str. 2, 142432 Chernogolovka, Russia; 3 Independent Researcher, 198504 Saint-Petersburg, Russia

**Keywords:** ion-selective electrodes, potentiometric sensitivity, QSPR, heavy metals sensing

## Abstract

While potentiometric, plasticized membrane sensors are known as convenient, portable and inexpensive analytical instruments, their development is time- and resource-consuming, with a poorly predictable outcome. In this study, we investigated the applicability of the QSPR (quantitative structure–property relationship) method for predicting the potentiometric sensitivity of plasticized polymeric membrane sensors, using the ionophore chemical structure as model input. The QSPR model was based on the literature data on sensitivity, from previously studied, structurally similar ionophores, and it has shown reasonably good metrics in relating ionophore structures to their sensitivities towards Cu^2+^, Cd^2+^ and Pb^2+^. The model predictions for four newly synthesized diphenylphosphoryl acetamide ionophores were compared with real potentiometric experimental data for these ionophores, and satisfactory agreement was observed, implying the validity of the proposed approach.

## 1. Introduction

Potentiometric sensors (ion-selective electrodes) are popular analytical devices for measuring ion concentrations (activities) in aqueous solutions. Unlike many other analytical methods, they are simple, in terms of instrumentation and portability, and not very expensive, yet quite accurate. These sensors also allow in-line and on-line measurements in automated mode. Such advantages have led to the wide application of potentiometric sensors in pharmaceutical [1], environmental [2], and food [3] analysis, and in numerous other fields.

Depending on the type of the sensitive membrane (glass, polymeric, polycrystalline) there are several varieties of ion-selective electrodes; however, the majority of modern studies in this field are devoted to sensors with plasticized polymeric membranes. This is due to the broad options for tuning the analytical performance of such membranes, by changing the active substance, the ion-exchanger, and the solvent-plasticizer [4]. Lipophilic ligands, or ionophores, represent the most important component of polymeric membranes, which is responsible for the selectivity and sensitivity of such sensors [5]. The search for novel effective ionophores, towards different ions, is a long and tedious process. It requires some initial assumptions on the possible structure of the ionophore, assessment of its synthetic availability, synthesis and purification of the candidate substance and, finally, the preparation of sensor membranes and their electrochemical characterization. As the overall sensor performance of ion-selective membrane depends on a complex interplay of multiple factors, it is often hard to predict in advance, and the resultant sensors may have insufficient characteristics for real analytical applications; thus, at the end of this chain of synthetic and experimental efforts, it may turn out that the properties of the sensor are not at all as good as originally thought.

Recently, it was shown that the Quantitative Structure-Property Relationship (QSPR) approach can be effective for in silico prediction of sensor properties based on an ionophore structure [6,7,8]. In the framework of this approach, the molecular structures of the chemical compounds are described by certain numbers: molecular descriptors, indicating the presence of particular atoms, bonds, their mutual positions in the structure and other structural properties [9]. These numbers are further related to the property of interest (e.g., toxicity of a substance) through mathematical modelling. If the model is statistically sound, this gives an opportunity for predicting the properties of novel substances for which the property of interest is not yet known. Obviously, there are certain limitations to this approach: typically, rather large datasets have to be modelled, in order to yield reasonable precision, and a certain level of structural similarity is expected between the substances from the training dataset and those to be predicted. QSPR has already been successfully applied to numerous problems, including drug discovery [10], toxicity evaluation [11], environmental problems [12], and material science [13,14].

Recent studies have shown that QSPR can be successfully applied to modelling the sensing properties of ionophore-based potentiometric sensors, including sensitivity [15] and selectivity [16,17] parameters. It has also been shown that similar approaches work well with potentiometric sensors, based on inorganic materials, like zeolites [18]. The report [16] demonstrated that prediction of the selectivity coefficients for newly synthesized carbonate ionophores is possible through QSPR, with reasonable precision. This study aims to extend the application of QSPR in the field of potentiometric sensors with novel tasks—we aim to predict the sensitivity of several new ionophores towards three heavy metal ions: copper, cadmium and lead. Although the QSPR models relating ionophore structures to the electrochemical response of the plasticized membrane sensors were described in the literature [15], they have never been applied to predicting the sensitivities of novel membrane sensors based on yet-unexplored ionophores. This study aims to fill this gap.

It has been found that several novel diphenylphosphoryl acetamides demonstrate increased extraction ability towards metal ions [19]. Our previous studies have shown that similar extraction agents can be successfully employed for developing polymer-plasticized potentiometric sensors for various metals [6,20]. In this work, we developed a QSPR model based on a literature data for potentiometric sensitivity towards Cu^2+^, Cd^2+^ and Pb^2+^; then, we predicted these values for four new diphenylphosphoryl acetamides ionophores; finally, we checked the accuracy of the predictions, through a traditional potentiometric experiment, by synthesizing sensor membranes with these ionophores and studying their response towards metal ions in aqueous solutions.

## 2. Materials and Methods

### 2.1. Dataset for QSPR Modelling

In order to make QSPR models for potentiometric sensitivity predictions we acquired the literature data on the response of sensors with PVC-plasticized membranes towards Cu^2+^, Cd^2+^ and Pb^2+^. The choice of these particular metals was dictated by their toxicity and by the pronounced interest in these elements, in environmental studies. The largest part of the dataset (35 structures, and the data on their potentiometric responses) was taken from [15]. In order to improve the training set towards phosphorus-containing ionophores, six substances (##36–41, Table S1, Supplementary Materials) were added to the dataset. The structures of the ionophores, their IUPAC names and the literature references can be found in Table S1 in the Supplementary Materials.

### 2.2. Molecular Descriptors

**Figure 1:**
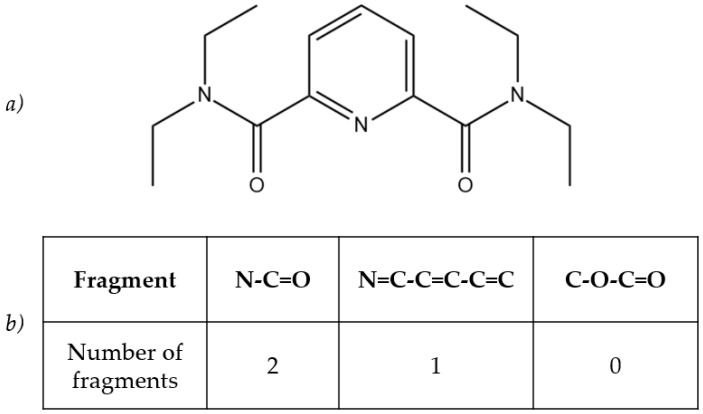
(**a**) The chemical structure of N2,N2,N6,N6-tetraethylpyridine-2,6-dicarboxamide. (**b**) Table with several fragments and their counts in the compound.

The structures of the ionophores were described with molecular descriptors. In this work, the ISIDA Substructure Molecular Fragments (SMF) were applied for this purpose. The method for obtaining molecular descriptors was similar to that in our previous article [16]. Briefly, ISIDA is a free software package for QSPR modelling, and can be applied for obtaining SMF [21]. Each SMF is a sequence of atoms in a chemical structure, that are connected with chemical bonds. A molecule can be represented as a graph; therefore, each SMF is the number of times a particular fragment appears in a molecule. These numbers are then collected into a matrix, where each row corresponds to a particular ionophore, and each column indicates the number of times a particular fragment appears in an ionophore. For example, in Figure 1a) there is a chemical structure of the compound from the Supplementary Materials. The table in Figure 1b) is a small piece of the total matrix with descriptors, and provides some fragments and the number of times these particular fragments can be found in the structure. Only the shortest path from one atom to another, with length from 2 to 9, atoms was considered. The resultant matrix of descriptors was 41 × 1095, where 41 was the number of ionophores in the training set, and 1095 was the number of their SMF molecular descriptors.

### 2.3. Regression Modelling

In order to relate the molecular descriptors of the ionophores with their potentiometric sensitivity towards copper, cadmium and lead, we employed the partial least squares regression method (PLS). This algorithm is widely applied for QSPR modelling [22,23], and it has gained broad popularity in chemometrics. The detailed description of the mathematics behind the PLS can be found in [24].

In the matrix form, the PLS model can be represented as follows:

(1) Y=XB

where *Y* is a column-vector of a dependent variable (potentiometric sensitivity towards particular metal for each particular ionophore); *X* is a matrix with independent variables (molecular descriptors for each particular ionophore); and *B* is a vector of regression coefficients. The absolute values of the regression coefficients obtained for each variable in *X* can be employed to judge on the importance of particular variables—molecular descriptors. One separate model was built for each of the three heavy metals.

The PLS models were cross-validated, for finding the optimal number of latent variables (a model parameter regulating the complexity of the model), using the leave-one-out approach, as the training dataset at hand was comparatively small (41 entries). The resultant models were evaluated with Root Mean Square Error (*RMSE*) and coefficient of determination R^2^. *RMSE* is a common metric for evaluation pf linear regression models; specifically, it allowed for evaluation of the difference between experimental and predicted values:

(2) $$RMSE=\sqrt{\frac{\sum_{i=1}^{n}{\left(y_{i}-\hat{y}\right)}^{2}}{n}}$$

where *y*_i_ was an experimental value; *ŷ* was a predicted value; and *n* was the number of samples in the model. R^2^ shows how strong was the power of the statistical relationship between the two variables: the values predicted by the model, and those known from the experiment.

### 2.4. Potentiometric Experiment

The developed PLS models were employed for prediction of potentiometric sensitivity towards copper, cadmium and lead, for four newly synthesized ionophores. The structures of the novel substances and their IUPAC names are shown in Table 1. Then, these substances were employed to synthesise plasticized polymeric sensor membranes. The synthesis and purification procedures of the ionophores are described in [25]. The sensor membranes were prepared using standard procedures: the weighted amounts of membrane components were dissolved in freshly distilled tetrahydrofuran (THF) in a glass beaker under stirring; these solutions were poured into 20 mm-in-diameter Teflon beakers, and left overnight to evaporate the solvent. The membranes contained 50 mmol/kg of an ionophore; 10 mmol/kg of chlorinated cobalt dicarbollide (Katchem, Prague, Czech Republic) as a cation-exchanger; 33% of high molecular weight poly(vinyl chloride) (Merck, Darmstadt, Germany); and 63–65% of *o*-nitrophenyl octyl ether as a solvent plasticizer (Merck, Darmstadt, Germany). Four sensor membranes were prepared, each one with the particular ionophore from Table 1. The resultant parent membranes were cut into disks, 8 mm in diameter, and attached to the PVC sensor bodies, using PVC–cyclohexanone solution. The resultant electrodes were filled with 0.01 M NaCl, and equipped with inner reference Ag/AgCl electrodes. Three identical sensors were prepared from each of the four membranes.

Multichannel digital mV-meter KHAN-32 (Sensor Systems LLC, St. Petersburg, Russia) was employed to register the potentials of the sensors in the aqueous samples. The standard Ag/AgCl electrode ESr-10101 (Izmeritel’naya Tekhnika, Moscow, Russia) was employed as a reference.

The potentiometric sensitivities of the sensors were measured in 10^−7^ to 10^−2^ M aqueous solutions of nitrates, of the following metals: cobalt, nickel, copper, zinc, cadmium and lead. The sensitivity values were calculated as the slopes of the linear parts of the sensor response curves. All the numerical values provided below represent an average, over at least three measurements, with three replicate sensors of each membrane composition.

## 3. Results and Discussion

**Figure 2:**
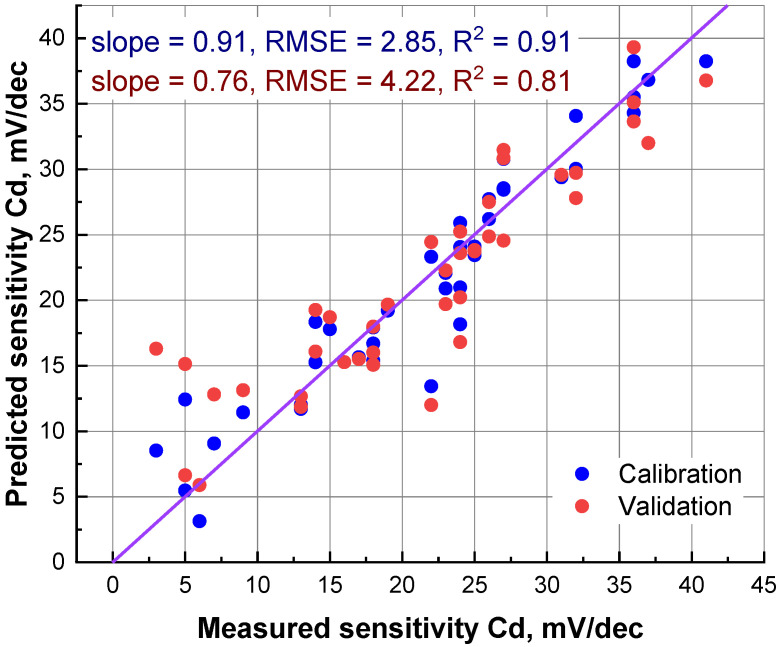
Measured vs. predicted scatterplot of cadmium sensitivity model, with four latent variables (LVs). The diagonal line shows the ideal correlation.

In the first stage of the experiment, we constructed three separate PLS models relating molecular descriptors of the ionophores in the training set with their potentiometric sensitivities to copper, cadmium and lead. Figure 2 shows a typical outcome of such modeling: a measured vs. predicted plot for cadmium sensitivity. One can see that a reasonably good correlation can be observed, and that the error in cross-validation was about 4 mV/dec. These results assume that the formalized ionophore description, using SMF, can be employed to predict the sensitivities of the corresponding plasticized polymeric sensor membranes.

**Figure 3:**
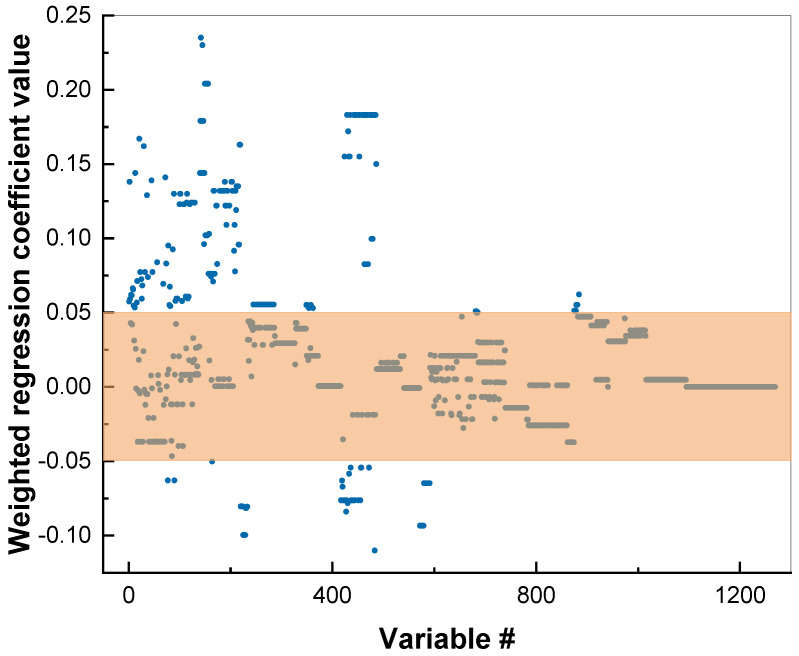
Regression coefficients plot for cadmium sensitivity PLS model, with marked area of fragments with small contributions. *X*-axis indicates the number of descriptor (variable).

The values of the regression coefficients in PLS modeling can be used to judge the importance of the molecular fragments for the particular target property. The plot of the regression coefficients is a convenient way to visualize these values. The weighted regression coefficients are calculated from the weighed data matrix with descriptors in the course of PLS regression; a brief explanation can be found in the Supplementary Materials. Figure 3 shows this plot for a cadmium sensitivity PLS model: the *X*-axis indicates the number of the descriptor, while the *Y*-axis indicates the numerical value of the particular regression coefficient for each descriptor. The higher the absolute value of the regression coefficient, the more important was the contribution of the corresponding descriptor. It can be seen that some of the descriptors did not contribute significantly to the model, and thus may have carried only noise. In order to optimize the model, we eliminated all the variables (descriptors) within the marked area of Figure 3, having small regression coefficient values between -0.05 and +0.05. Getting rid of the irrelevant variables led to the improvement of the models. A similar procedure was performed for copper and lead PLS models, and the resultant statistics are shown in Table 2. The ‘measured vs. predicted’ plots for copper and lead can be found in Figures S1 and S2 (Supplementary Materials).

It can be seen that the worst model performance was observed for lead, where the *RMSE* in cross-validation was more than 7 mV/dec. Obviously, such a model was suitable rather for qualitative assessment of the ionophores in terms of their suitability to produce lead sensors, than to a numerical prediction. Somewhat better results were obtained in the case of copper; however, they were still of semi-quantitative nature, in terms of prediction accuracy.

**Figure 4:**
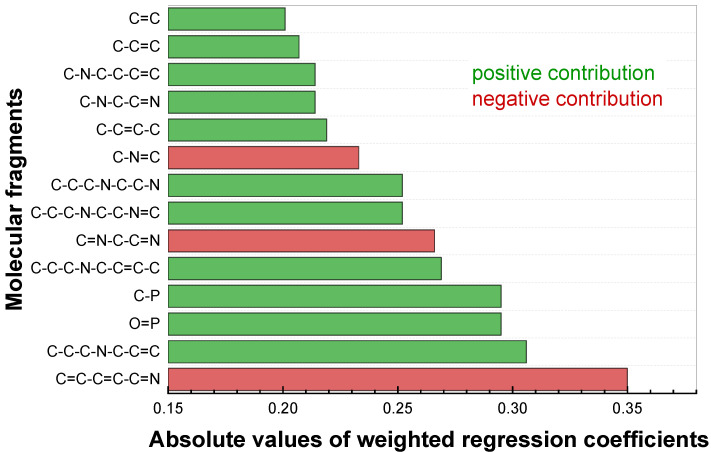
Fragments with high contributions to cadmium sensitivity.

An important indicator of the QSPR model validity was the compliance of the important molecular fragments with chemical intuition. Figure 4 shows the particular fragments that were important in characterizing sensitivity towards cadmium. They were selected based on the condition that they occurred in at least five structures, to avoid occasional correlations. When constructing the diagrams, we omitted the fragments with hydrocarbon chains at the ends, if the nested fragments had similar contributions. As an example of such fragments, one can consider the pair C-C-P-C=C-C and C-C-P-C=C. Figures S3 and S4 (Supplementary Materials) show these plots for copper and lead correspondingly.

**Figure 5:**
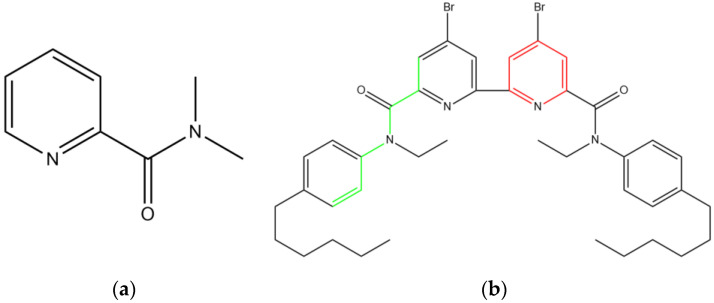
(**a**) N,N-dimethylpicolinamide fragment; (**b**) 4,4′-dibromo-N6,N6′-diethyl- -N6,N6′-bis(4-hexylphenyl)-[2,2′-bipyridine]-6,6′-dicarboxamide with highlighted C-C-C-N-C-C=C (green) and C=C-C=C-C=N (red) fragments.

An inspection of the plot in Figure 4 reveals that the fragments with the greatest contribution to cadmium sensitivity include a phosphorus atom or N,N-dimethylpicolinamide fragment (Figure 5a). Unlike copper sensitivity (Figure S3), fragments of the structure (C=C-C=C-C=N, C-N=C) mostly have a negative contribution. The greatest sensitivity to cadmium in the training dataset (41 mV/dec) had 4,4′-dibromo-N6,N6′-diethyl-N6,N6′-bis(4-hexylphenyl)-[2,2′-bipyridine]-6,6′-dicarboxamide. The chemical structure of this compound is given in Figure 5b. The compound contained fragments with both positive (highlighted in green) and negative (highlighted in red) contributions. In general, the identified important structural fragments followed the chemical logic of complex formation: the ionophores with picolinamide fragment (Figure 4a) showed pronounced sensitivity towards cadmium when employed in plasticized polymeric membranes of potentiometric sensors (see also Table S1). The same held for the green fragment in Figure 5b, which represented the part of picolinamide, although the absence of an oxygen atom in the green fragment looks questionable. Another discrepancy with experimental observations is the red fragment in Figure. 4b—generally, the presence of a pyridine structural unit was observed for the ionophores with both substantial and average Cd^2+^ sensitivity. We believe these discrepancies can be attributed to the rather small size of the employed dataset (41 ionophore only), while traditional QSPR studies normally involve hundreds or thousands of entries. It must be pointed out that, in cases of potentiometric experiments, the construction of such a large dataset is hardly possible, due to the differences in experimental protocols employed by the researchers to study potentiometric sensors (membrane compositions, sensitivity calculations, pH of the samples).

The obtained PLS models were applied to the prediction of potentiometric sensitivities for four new ionophores (Table 1). The chemical structures of these compounds were analyzed with a previously optimized set of molecular fragments. Then, these four rows of molecular descriptors served as inputs for the three PLS models, to predict the sensitivity to each of the metal cations. The results are shown in Table 3.

**Figure 6:**
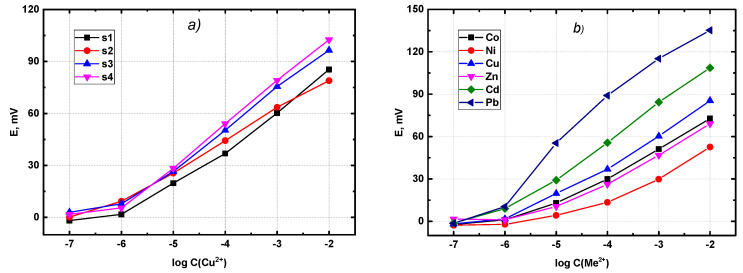
(**a**) Potentiometric response curves observed in copper solutions with four different types of sensors. (**b**) Potentiometric response curves for various metals observed with the sensor based on substance 1.

In order to check the validity of the QSPR predictions, a real potentiometric experiment was performed, including synthesis and electrochemical measurements with plasticized polymeric sensor membranes. Figure 6 shows the typical response curves obtained in aqueous solutions of metal nitrates.

It must be pointed out that we also explored the response of the newly synthesized sensors towards nickel, cobalt and zinc, as well as to several other metals. The main reason for limiting the list of cations in the QSPR modelling to copper, cadmium and lead was the availability of the corresponding literature data. The construction of the initial descriptor matrix required each substance from the database to be characterized with respect to its response towards all ions of interest. As some of the ligands in our database were not studied in sensors for nickel, cobalt and zinc, we had to limit the list of the metals. In accordance with previous studies, the substances with pronounced extraction ability towards lanthanides demonstrated a significant potentiometric response towards d-elements in aqueous solutions. The complete table, with all obtained sensitivities for all studied ionophores, can be found in the Supplementary Materials (Table S2), where a certain structural dependence of the sensitivity values for cobalt and nickel can be observed. The longer the linking alkyl chain between two nitrogen atoms in the ligand (compare substances 1, 2 and 3) the higher was the response to cobalt and nickel. At the same time, this trend was not pronounced for the rest of the metals. The behaviour of substance 4 with piperazine linker is different from the trends observed for the alkyl-linked substances. The supposed mechanism was believed to be the same for all studied substances, and it was based on a complex formation between the ligand and the target ion, using the phosphine oxide oxygen and keto atoms, with certain impact from nitrogen atoms; however, no further assumptions would be substantiated without dedicated studies. The responses towards cadmium and lead were close to the theoretical values for doubly charged cations (29.5 mV/dec), while the sensitivities to other cations were sub-Nernstian. Figure 6b shows the typical response curves (sensor sensitivity) obtained for the ionophore 1. One can see that the smallest response was observed for cobalt, nickel and zinc, while the highest one was for lead ions. This behaviour was typical for all other ligands (Table S2).

The comparison of the QSPR predictions and the potentiometric experiment is given in Table 3. In most of the cases, one can observe a very good correspondence between the model prediction and the real potentiometric experiment. It was especially good in the case of cadmium, while in the case of lead, all the predicted values were systematically lower than the experiment; however, the relative order in sensitivity increase from copper to lead was well-followed by the model predictions.

## 4. Conclusions

We have demonstrated that the QSPR model, trained on the literature data on potentiometric sensitivity of plasticized polymeric membranes based on different ionophores, can be successfully employed for in silico prediction of the potentiometric behavior of new potential ionophores, without performing real synthesis and characterization of sensor membranes. The validity of QSPR model predictions for sensitivities towards copper, cadmium and lead, for four newly synthesized diphenylphosphoryl acetamide ionophores, was checked, through the real potentiometric experiment, employing these ionophores. The observed correspondence between the model predictions and real experimental sensitivity values implied the high practical potential of the QSPR in developing novel plasticized membrane sensors with the required characteristics.

## Figures and Tables

**Table 1:** Chemical structures and IUPAC names of the diphenylphosphoryl acetamides.

	Chemical Structure	IUPAC Name
1	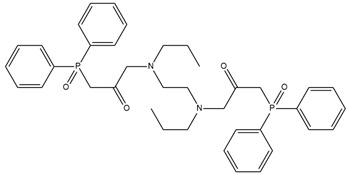	3,3′-(butane-1,4-diylbis(octylazanediyl))bis(1-(diphenylphosphoryl)propan-2-one)
2	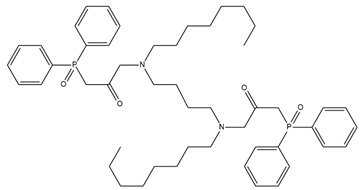	3,3′-(butane -1,4-diylbis(octylazanediyl))bis(1-(diphenylphosphoryl)propan-2-one)
3	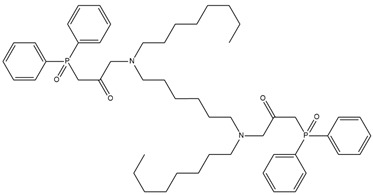	3,3′-(hexane-1,6-diylbis(octylazanediyl))bis(1-(diphenylphosphoryl)propan-2-one)
4	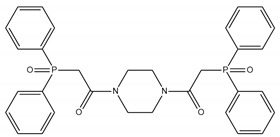	1,1′-(piperazine-1,4-diyl)bis(2-(diphenylphosphoryl)ethanone)

**Table 2:** QSPR model parameters for copper, cadmium and lead.

		Slope	*RMSE*	R^2^
Cu^2+^	calibration	0.86	4.29	0.86
	validation	0.64	6.88	0.66
Cd^2+^	calibration	0.91	2.85	0.91
	validation	0.76	4.22	0.81
Pb^2+^	calibration	0.95	2.60	0.95
	validation	0.55	7.49	0.64

**Table 3:** The experimental and predicted sensitivity. The standard deviation values for experimental data obtained over three identical sensors in three replicated measurements did not exceed ±1.5 mV/dec.

	Cu^2+^		Cd^2+^		Pb^2+^	
	Experimental	Predicted	Experimental	Predicted	Experimental	Predicted
1	20.3	20.9	24.6	24.7	32.9	29.7
2	18.3	16.4	22.1	22.5	31.7	29.2
3	21.1	15.3	24.7	22.5	34.6	29.1
4	23.4	19.4	23.0	23.1	34.9	27.2

## Data Availability

Not applicable.

## Supplementary Materials

The following supporting information can be downloaded at: https://www.mdpi.com/article/10.3390/membranes12100953/s1. Table S1: Structures, sensitivity and literature sources of the ionophores that were collected for the database. Figure S1: measured vs. predicted scatterplot of copper sensitivity model, with the plotted line of the ideal dependence. Figure S2: measured vs. predicted scatterplot of lead sensitivity model, with the plotted line of the ideal dependence. Figure S3: fragments with high contributions to copper sensitivity. Figure S4: piece of chemical structure with highlighted C-N-C-C-N=C molecular fragment. Figure S5: fragments with high contributions to lead sensitivity. Table S2: potentiometric sensitivities of the sensors towards metal cations (±1 mV/dec) [15,26].
