# Supplementary material for: Predicting the Potentiometric Sensitivity of Membrane Sensors Based on Modified Diphenylphosphoryl Acetamide Ionophores with QSPR Modeling
Source: Membranes (Basel). 2022 Sep 29;12(10):953. doi: 10.3390/membranes12100953 (PMC9611910; doi:10.3390/membranes12100953)
Supplement: Supplementary file 1 [file membranes-12-00953-s001.zip › membranes-1933226-supplementary.pdf]

Supplementary Material

Predicting potentiometric sensitivity of membrane sensors based on modified diphenylphosphoryl acetamide ionophores with QSPR modeling

Nadezhda Vladimirova [1], Elena Puchkova[1], Dmitry Dar'in[1], Alexander Turanov[2], Vasily Babain[3] and Dmitry Kirsanov [1*]

[1] Institute of Chemistry, Saint-Petersburg State University, Peterhof, Universitetsky Prospect, 26, Saint-Petersburg, 198504, Russia
[2] Yu. A. Ossipyan Institute of Solid-State Physics, Russian Academy of Sciences, Chernogolovka, Moscow oblast, Academician Osipyan str. 2, 142432 Russia
[3] independent researcher, Saint-Petersburg, Russia.

* Correspondence: d.kirsanov@gmail.com

Table S1. Structures, sensitivity and literature sources of the ionophores that are collected for the database

#	Structure of the ionophore	Sensitivity, mV\dec			Reference
		Cd^{2+}	Cu^{2+}	Pb^{2+}	
1	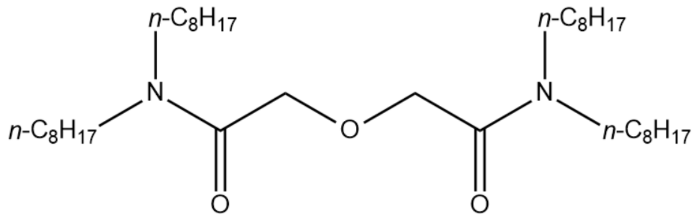	9	5	30	[S1]
2	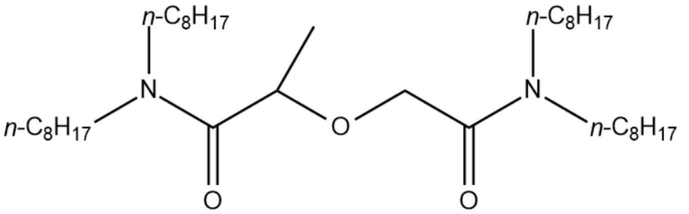	13	12	24	[S1]
3	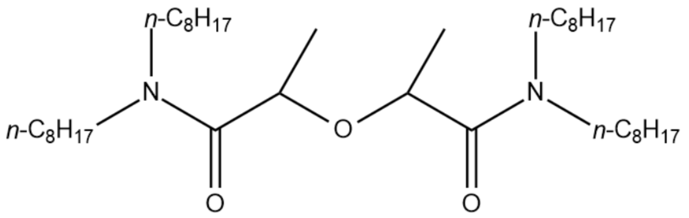	13	9	18	[S1]

4	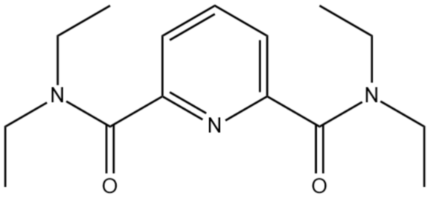	14	25	27	[S1]
5	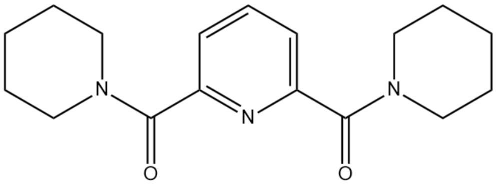	14	23	26	[S1]
6	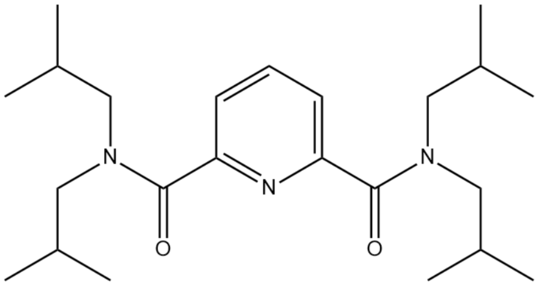	27	34	51	[S1]
7	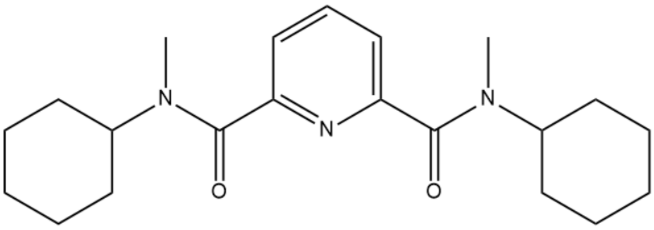	21	31	37	[S1]
8	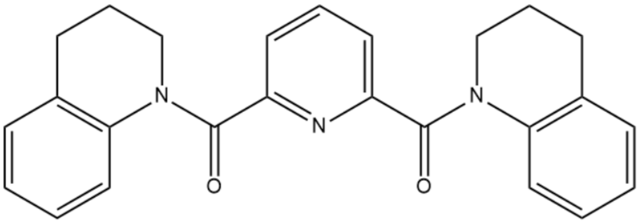	26	34	34	[S1]
9	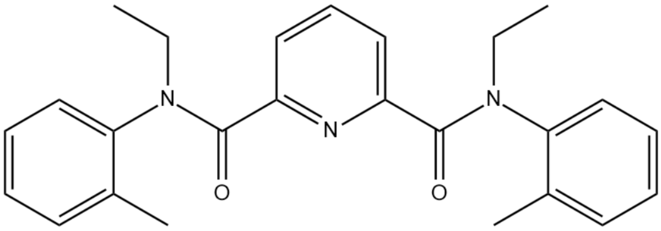	32	43	44	[S1]
10	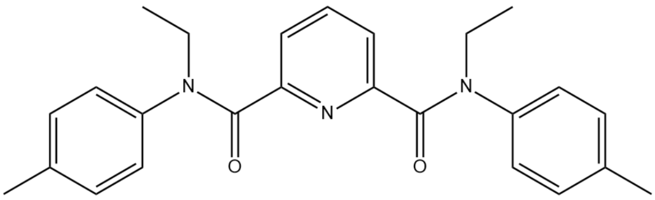	32	43	45	[S1]

11	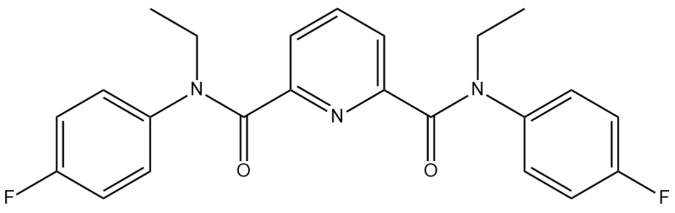	19	37	37	[S1]
12	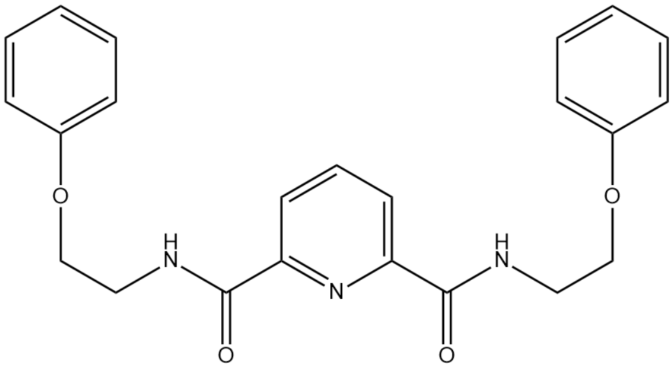	23	28	28	[S1]
13	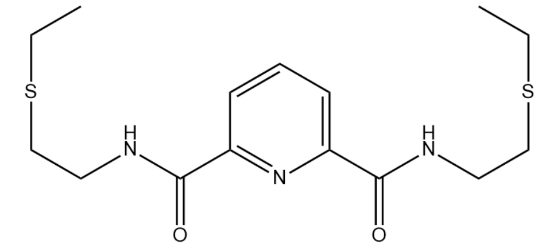	22	27	28	[S1]
14	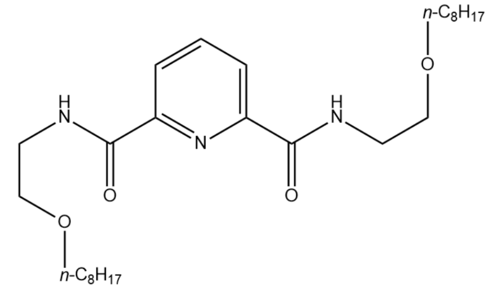	24	31	31	[S1]
15	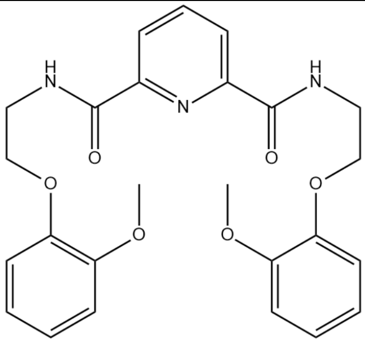	25	28	29	[S1]

16	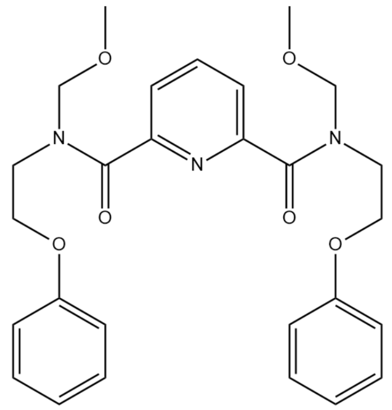	22	25	42	[S1]
17	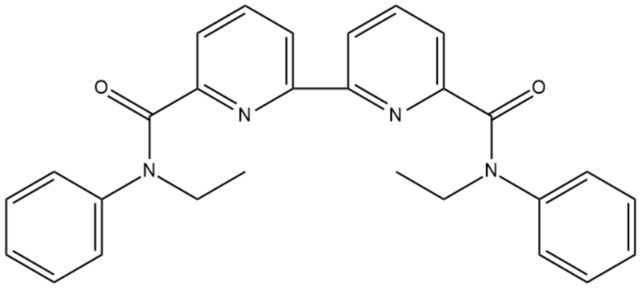	36	31	24	[S1]
18	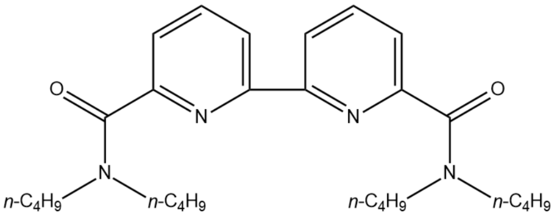	37	39	26	[S1]
19	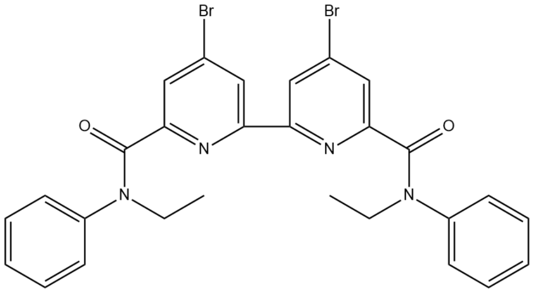	36	26	28	[S1]
20	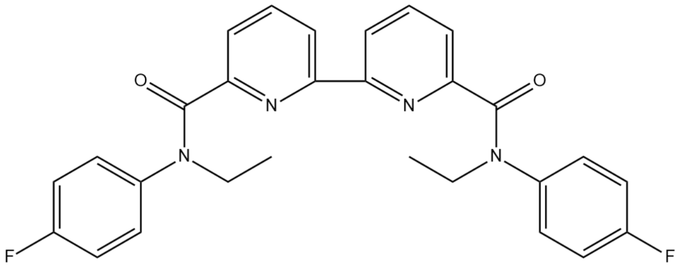	31	30	23	[S1]
21	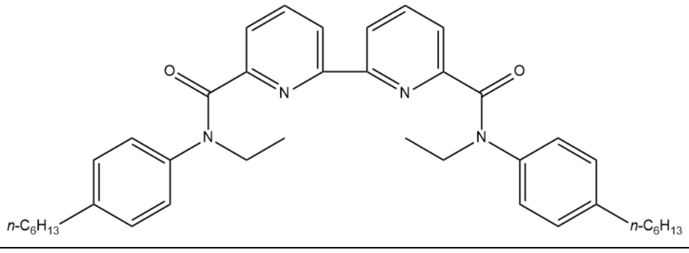	36	34	23	[S1]

22	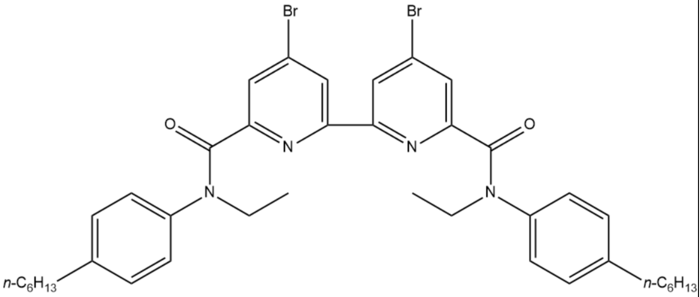	41	31	27	[S1]
23	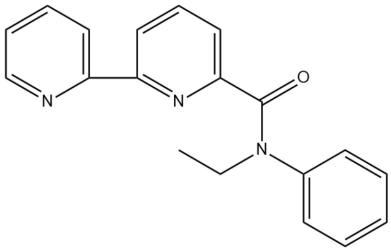	3	-12	0	[S1]
24	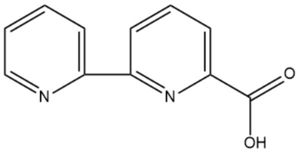	6	1	0	[S1]
25	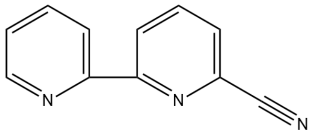	5	-10	3	[S1]
26	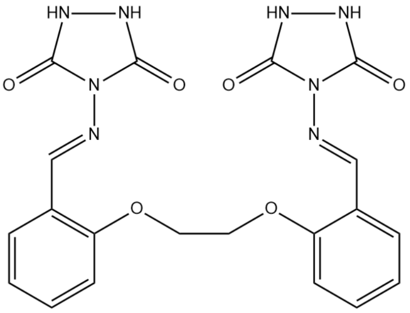	16	15	24	[S1]
27	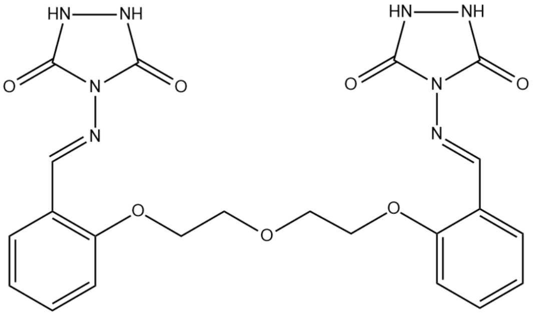	15	15	24	[S1]
28	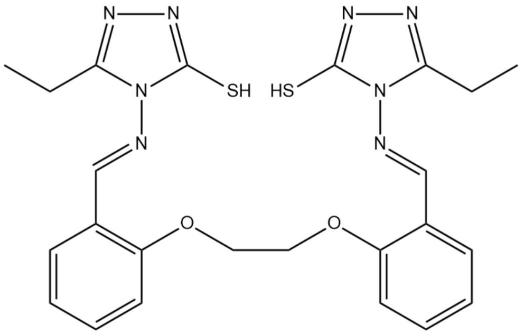	17	19	25	[S1]

29	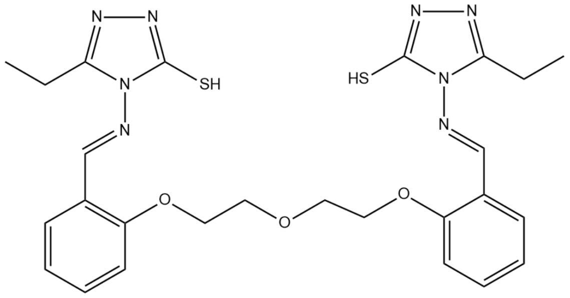	24	34	24	[S1]
30	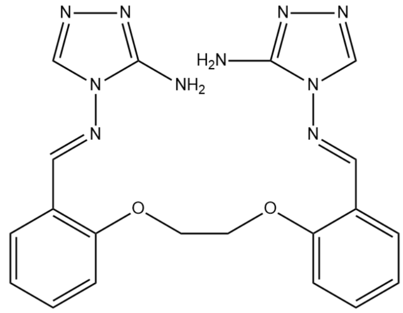	18	28	27	[S1]
31	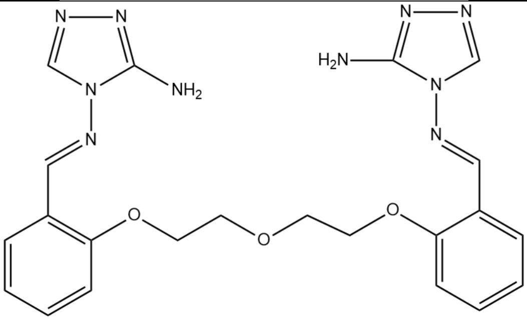	18	20	28	[S1]
32	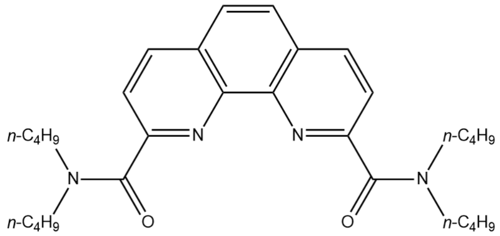	26	24	31	[S1]
33	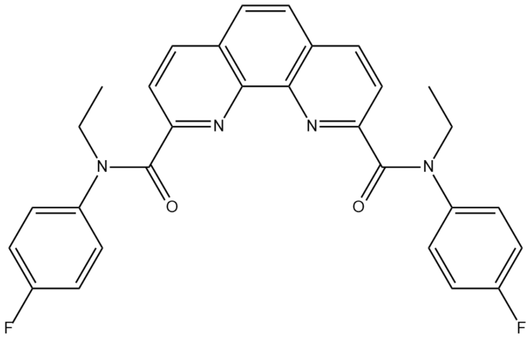	27	23	26	[S1]
34	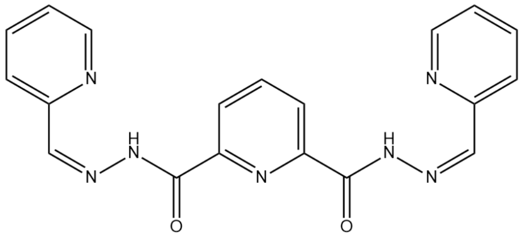	7	0	0	[S1]

35	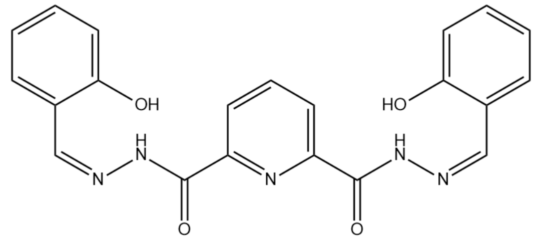	5	0	4	[S1]
36	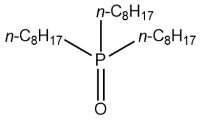	18	9	9	[S2]
37	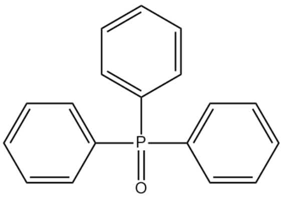	-10	5	-20	[S2]
38	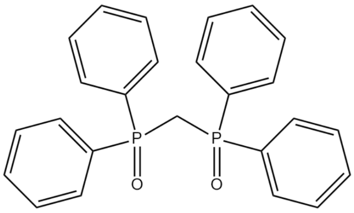	23	30	38	[S2]
39	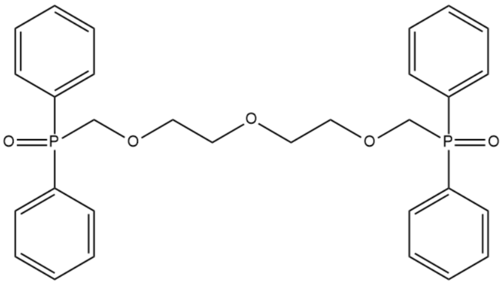	25	30	33	[S2]
40	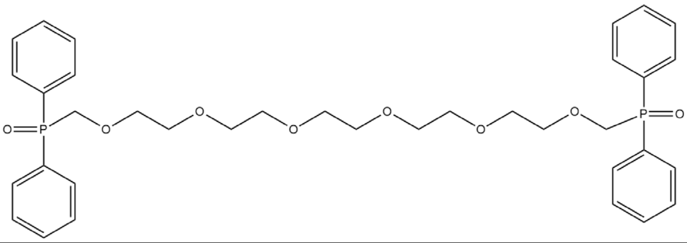	27	26	40	[S2]
41	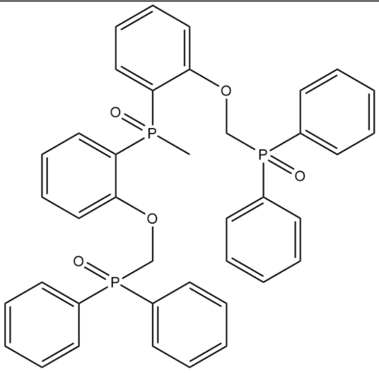	24	20	16	[S2]

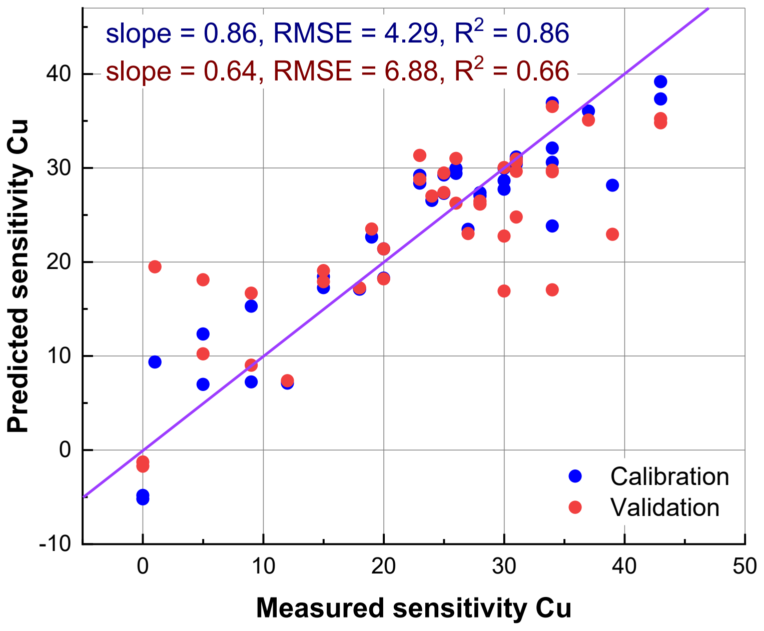

Figure S1. Measured vs predicted scatterplot of copper sensitivity model (two latent variables) with the plotted line of the ideal dependence.

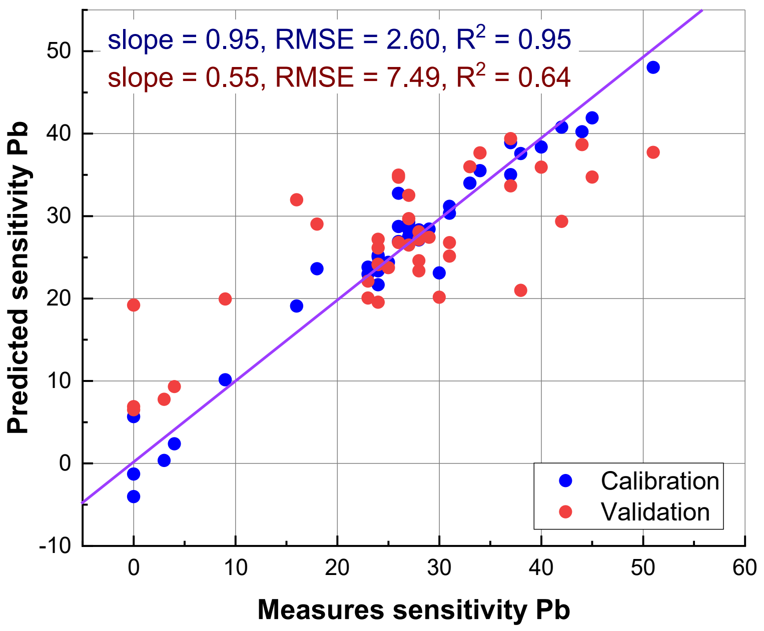

Figure S2. Measured vs predicted scatterplot of lead sensitivity model (six latent variables) with the plotted line of the ideal dependence.

The procedure for calculating weighted regression coefficients:

The weighted regression coefficients are calculated from the weighed data matrix with descriptors in the course of PLS regression. The detailed description can be found in ref [25]. Briefly, we decompose both X (descriptors) and Y (target sensitivity values) into latent variable space:

$X = TP^T + E;$

$Y = UQ^T + F$

Then we calculate the weighted loadings matrix:

$W = \max(\mathrm{cov}(T,U)),$

and finally we calculate regression coefficients:

$B = W(P^TW)^{-1}Q^T$

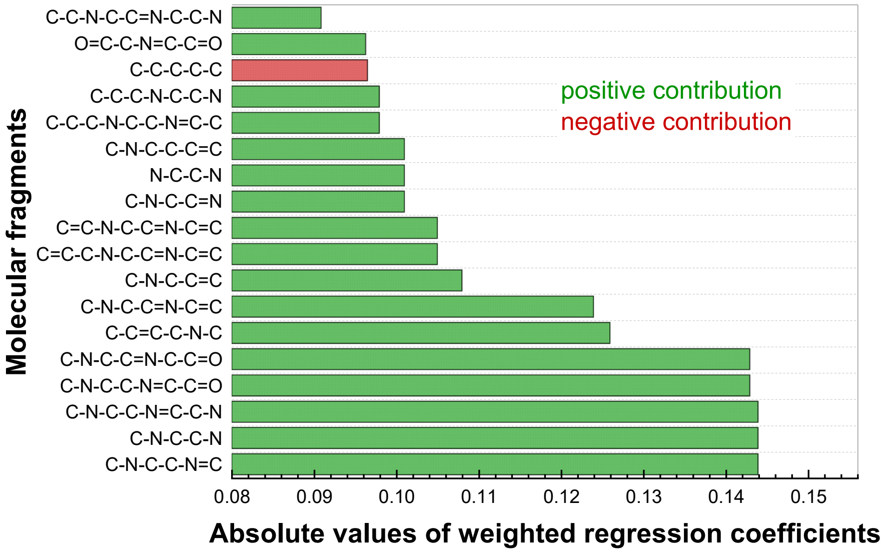

Figure S3. Fragments with high contributions to copper sensitivity

The fragments with the greatest positive contribution could be found in the following chemical structural piece (Fig. S4). Among these fragments are C-N-C-C-N=C and C-N-C-C-N, C-C=C-C-N-C and C-N-C-C=C. The fragment C-N-C-C-N=C with the greatest regression coefficient value is highlightened in the Fig. S4. This structural piece is part of compounds #17-23 in the database with high copper sensitivity.

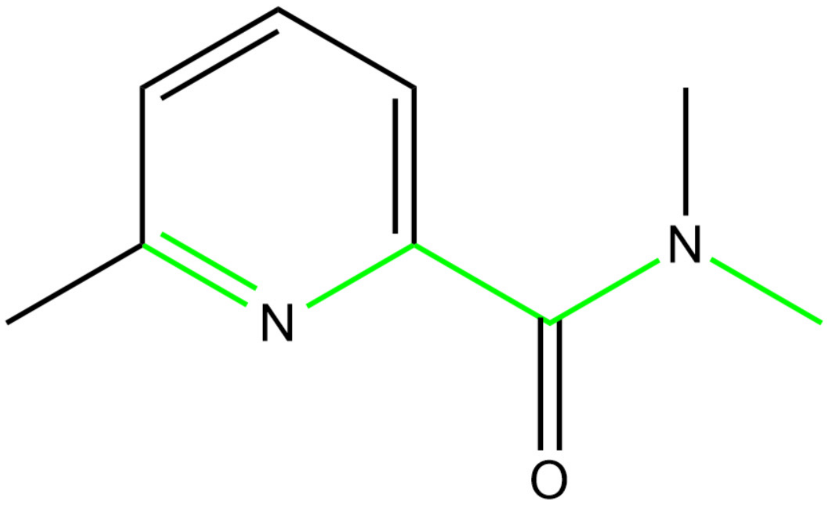

Figure S4. Piece of chemical structure with highlightened C-N-C-C-N=C molecular fragment.

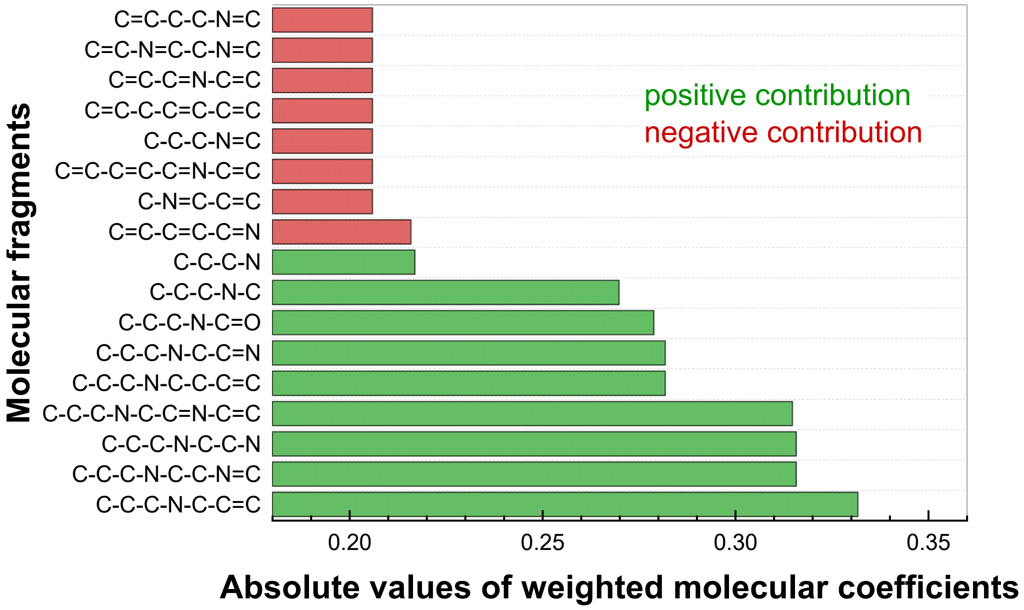

Figure S5. Fragments with high contributions to lead sensitivity

N2,N2,N6,N6-tetraisobutylpyridine-2,6-dicarboxamide (ionophore #6 in the Table S1) has the greatest sensitivity to lead (51 mV/dec). It has several fragments with significant positive contribution such as C-C-C-N-C-C-N=C, C-C-C-N-C-C-N, C-C-C-N-C-C=N-C=C, C-C-C-N-C-C=N and C-C-C-N-C=O and a couple of fragments (C=C-C=C-C=N, C-N=C-C=C) with significant negative contribution.

Table S2. Potentiometric sensitivities of the sensors towards metal cations (±1.5 mV/dec).

Sensor	Co^{2+}	Ni^{2+}	Cu^{2+}	Zn^{2+}	Cd^{2+}	Pb^{2+}
**1**	18	13	21	18	25	33
**2**	20	16	18	15	22	32
**3**	22	18	21	19	25	34
**4**	18	16	23	20	23	35

Supplementary References

S1. Soloviev, V.; Kirsanov, D. et al. QSPR modeling of potentiometric sensitivity towards heavy metal ions for polymeric membrane sensors. *Sens. Actuators B Chem.*, **2019,** *301.* doi:10.1016/j.snb.2019.126941

S2. Kirsanov, D., Khaydukova, M., Tkachenko, L., Legin, A. and Babain, V. Potentiometric Sensor Array for Analysis of Complex Rare Earth Mixtures. *Electroanalysis,* **2012,** *24,* pp. 121-130. doi: 10.1002/elan.201100439
